# Supplementary material for: XRRA1 acts as a molecular brake on radiation-induced DNA damage signaling and immunogenic cell death in tumor cells
Source: bioRxiv. 2026 Apr 24:2026.04.21.719372. Preprint. [Version 1] doi: 10.64898/2026.04.21.719372 (PMC13131765; doi:10.64898/2026.04.21.719372)
Supplement: Supplement 1 [file NIHPP2026.04.21.719372v1-supplement-1.pdf]

## Supplementary Figure legends

A.

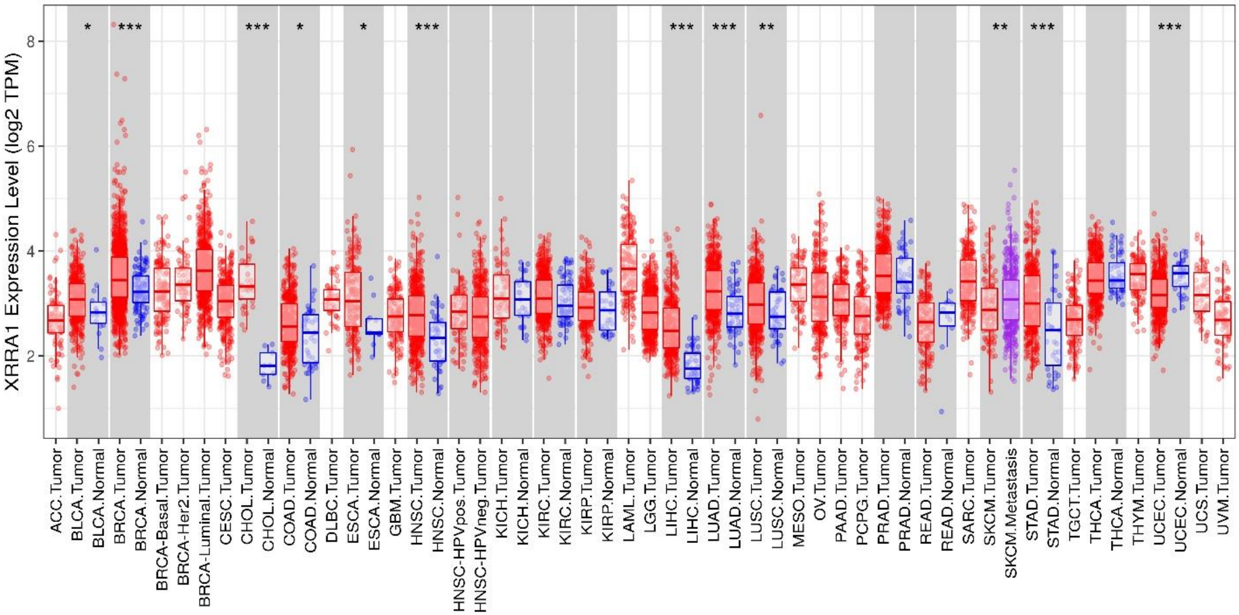

bioRxiv preprint doi: https://doi.org/10.64898/2026.04.21.719372; this version posted April 24, 2026. The copyright holder for this preprint (which was not certified by peer review) is the author/funder, who has granted bioRxiv a license to display the preprint in perpetuity. It is made available under aCC-BY 4.0 International license.

B.

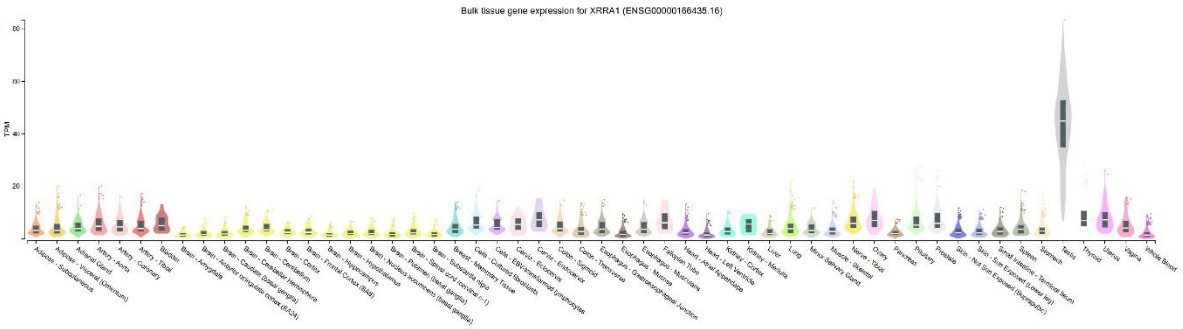


C.

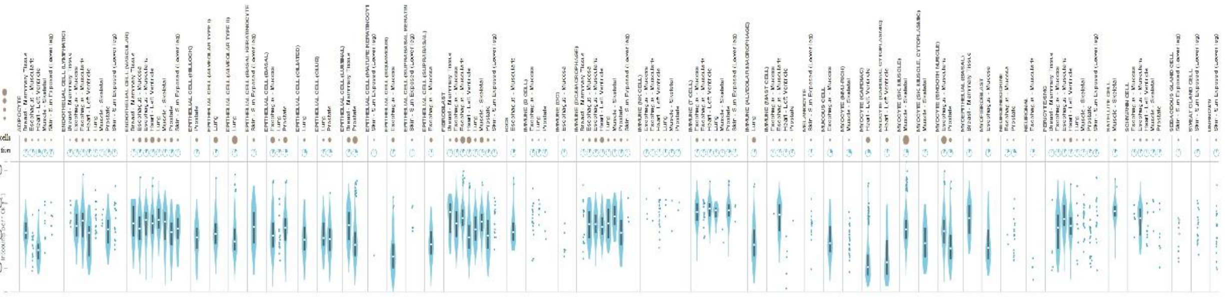


D.

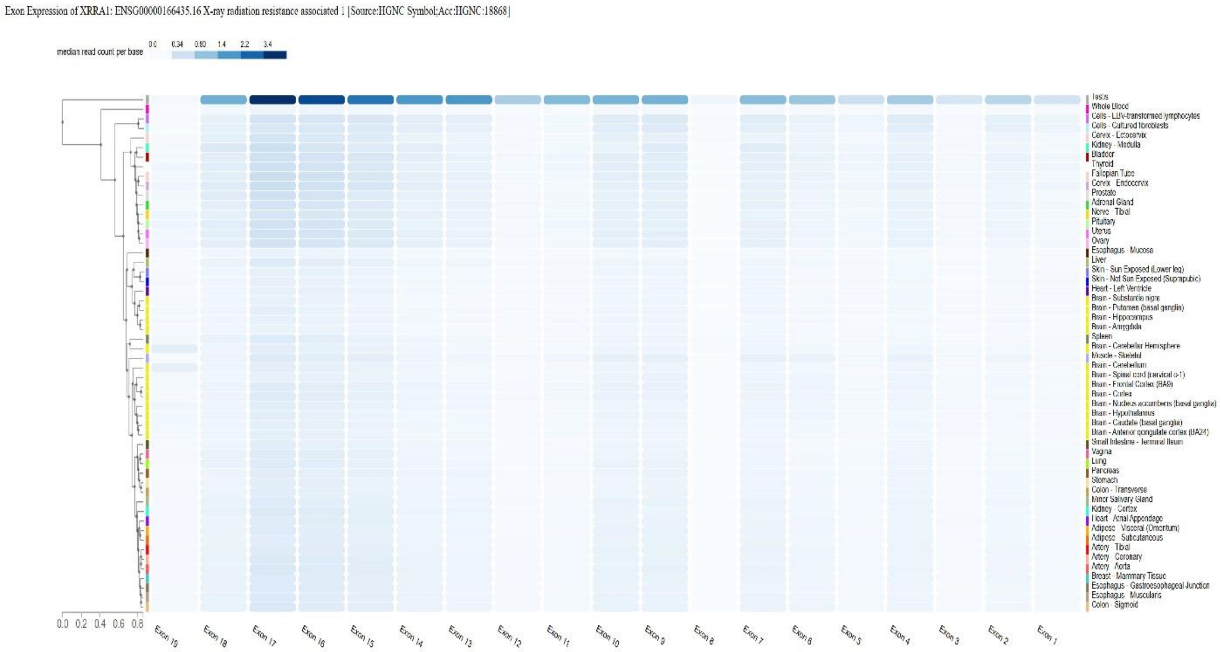

bioRxiv preprint doi: https://doi.org/10.64898/2026.04.21.719372; this version posted April 24, 2026. The copyright holder for this preprint (which was not certified by peer review) is the author/funder, who has granted bioRxiv a license to display the preprint in perpetuity. It is made available under aCC-BY 4.0 International license.

E.

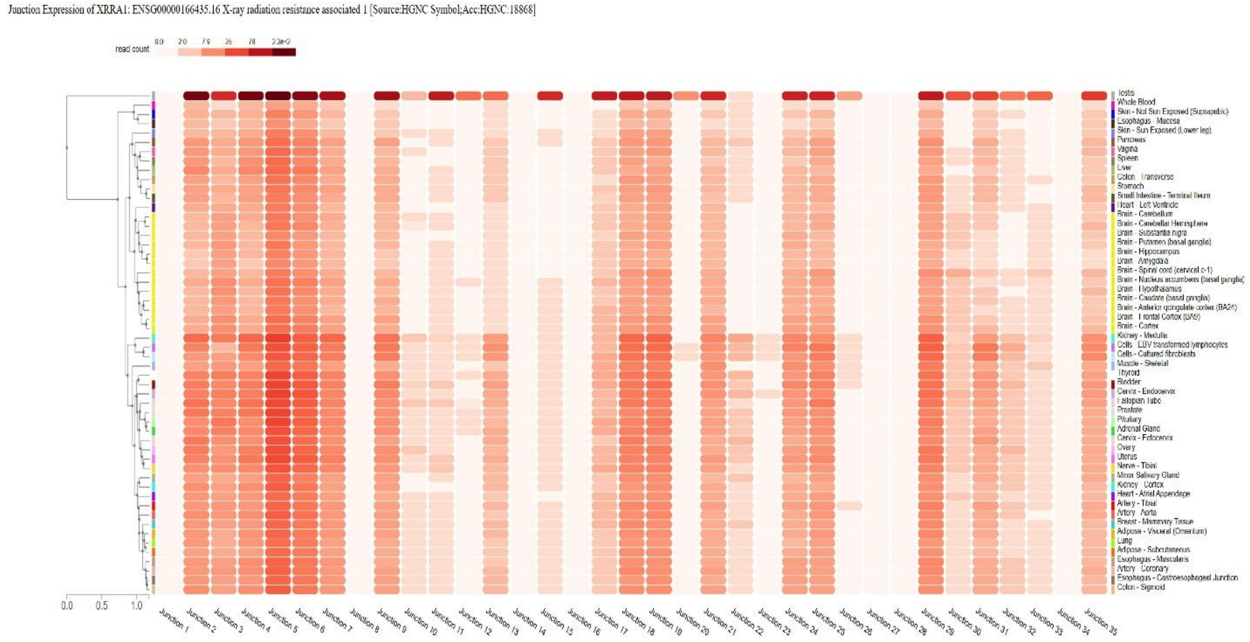


F.

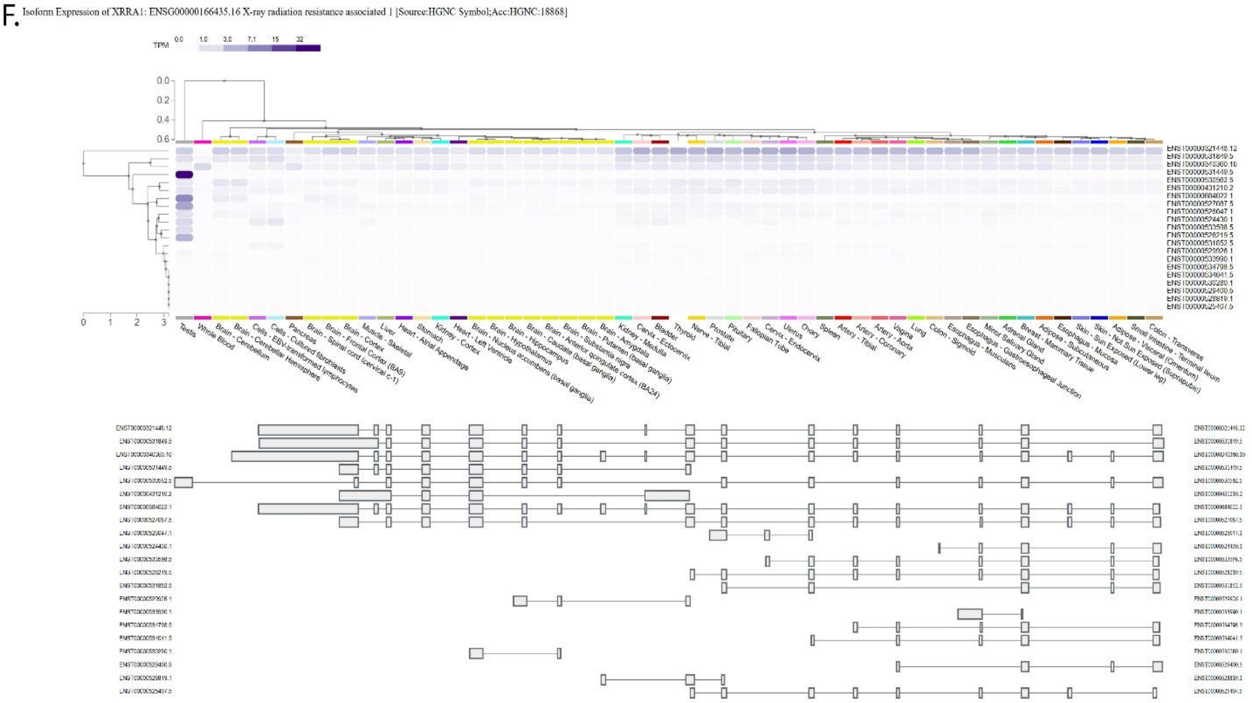

bioRxiv preprint doi: https://doi.org/10.64898/2026.04.21.719372; this version posted April 24, 2026. The copyright holder for this preprint (which was not certified by peer review) is the author/funder, who has granted bioRxiv a license to display the preprint in perpetuity. It is made available under aCC-BY 4.0 International license.

G.

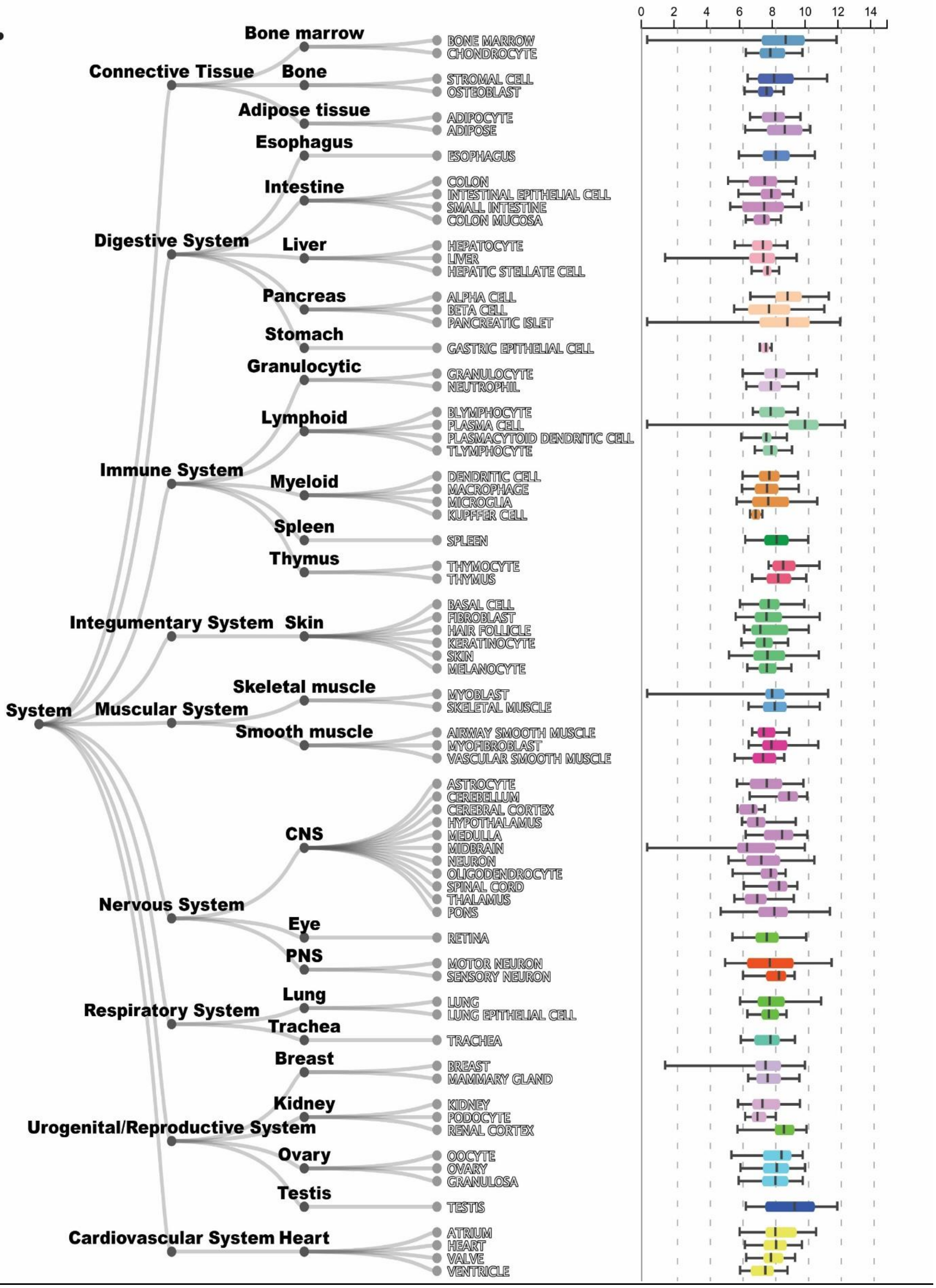

bioRxiv preprint doi: https://doi.org/10.64898/2026.04.21.719372; this version posted April 24, 2026. The copyright holder for this preprint (which was not certified by peer review) is the author/funder, who has granted bioRxiv a license to display the preprint in perpetuity. It is made available under aCC-BY 4.0 International license.

H.

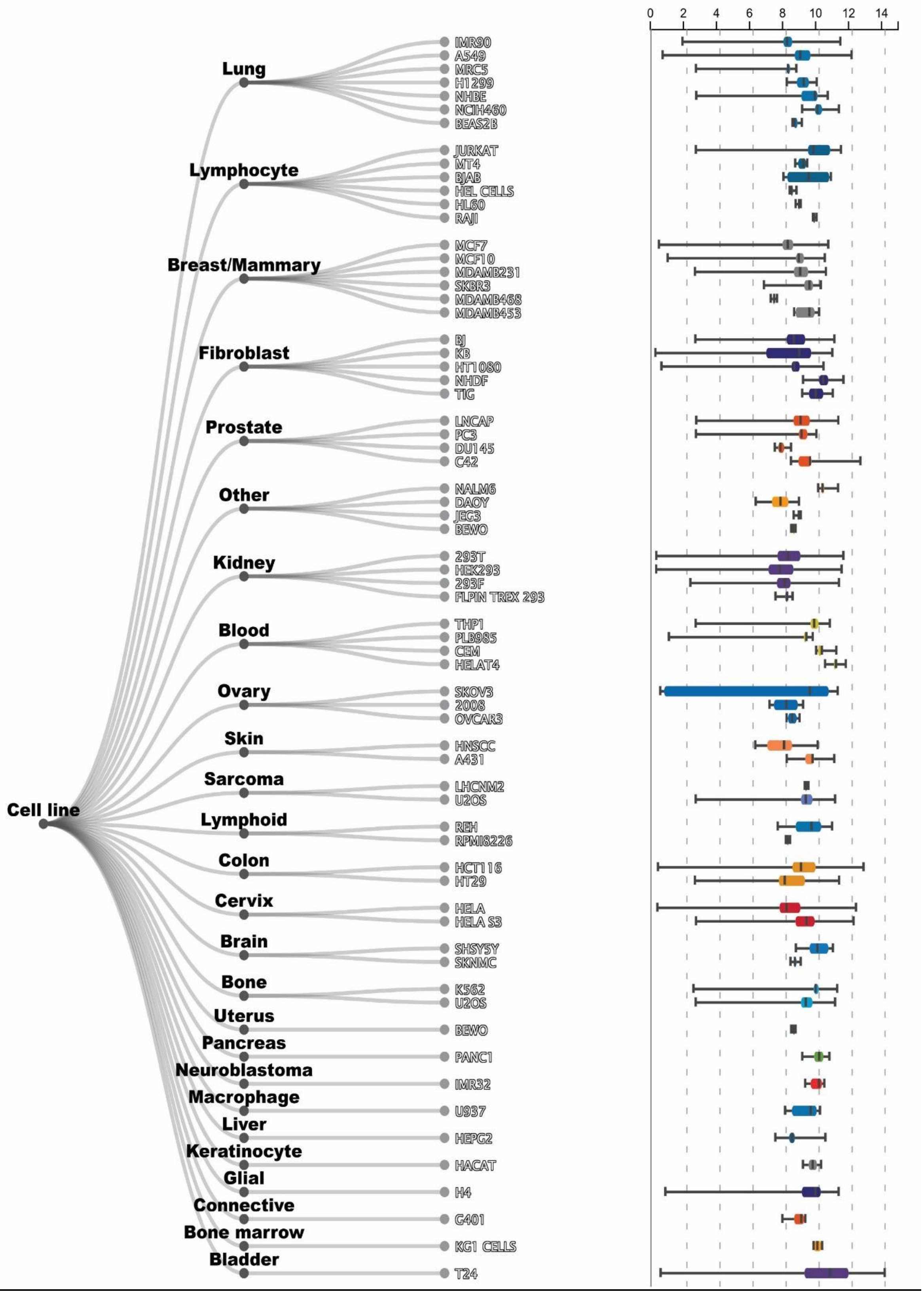

bioRxiv preprint doi: https://doi.org/10.64898/2026.04.21.719372; this version posted April 24, 2026. The copyright holder for this preprint (which was not certified by peer review) is the author/funder, who has granted bioRxiv a license to display the preprint in perpetuity. It is made available under aCC-BY 4.0 International license.

I.

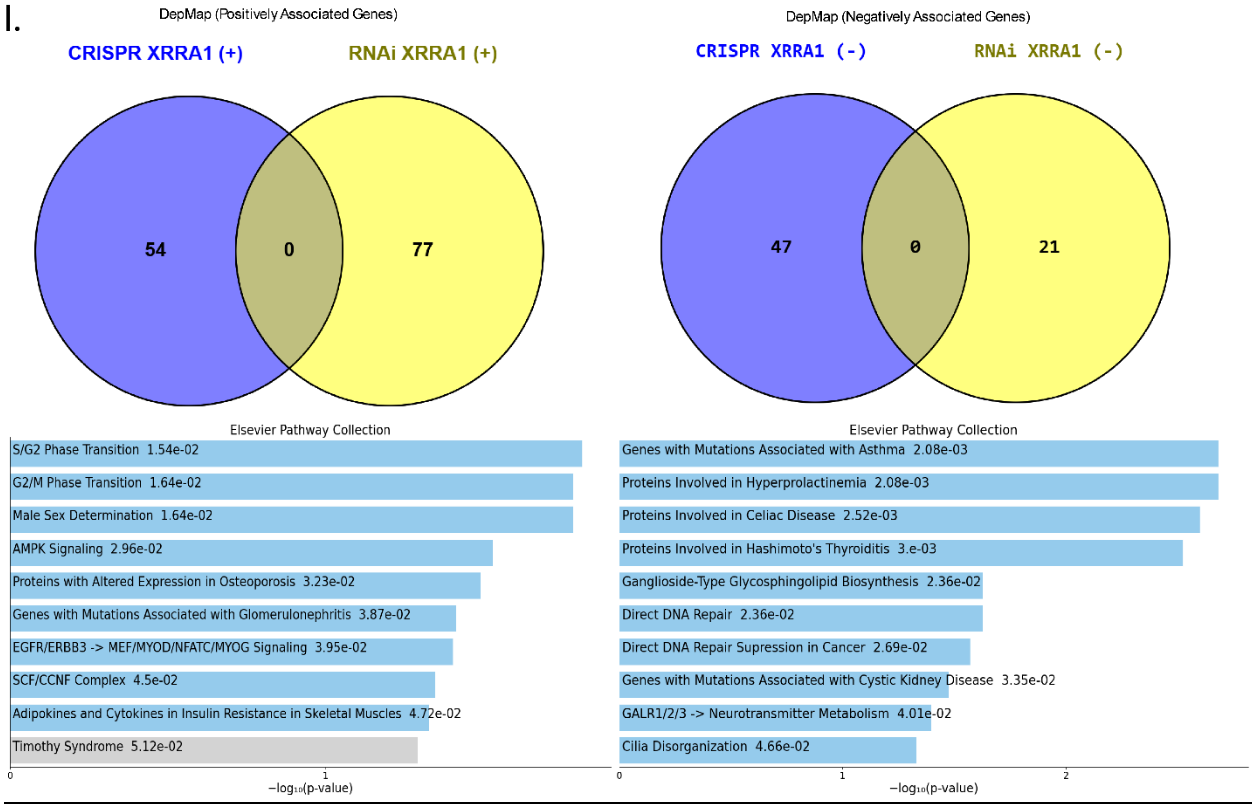


**Supplementary Figure 1: Expression landscape, transcript organization and dependency context of XRRA1. S1A,** Pan-cancer comparison of XRRA1 mRNA expression across tumor and normal tissues. Box plots with overlaid data points show XRRA1 expression levels (log2 TPM) in tumor tissues (red) and normal tissues (blue); metastatic melanoma samples are shown in purple, where available. Cancer-type abbreviations follow standard TCGA nomenclature. Asterisks indicate statistically significant differences between groups. **S1B,** Bulk normal-tissue expression profile of XRRA1 across human tissues. Violin plots show TPM distributions across tissues, indicating broad basal expression with marked enrichment in selected tissues, including testis. **S1C,** Single-cell expression atlas of XRRA1 across human tissues. The upper tracks indicate the total number of cells analyzed and the fraction of XRRA1-expressing cells for each annotated cell type, whereas the lower violin plots show the distribution of XRRA1 expression at single-cell resolution. **S1D,** Exon-level expression heat map of XRRA1 across normal tissues. Columns correspond to XRRA1 exons 1-19, and color intensity represents median read count per base. Tissues are hierarchically clustered according to exon-level expression patterns. **S1E,** Junction-level expression heat map of XRRA1 across normal tissues. Columns correspond to splice junctions 1–35, and color intensity represents junction read count. Tissues are hierarchically clustered according to splice-junction usage. **S1F,** Isoform expression landscape of XRRA1 across normal tissues. The upper heat map shows clustered isoform-level expression (TPM) across tissues, and the lower panel shows the exon–intron architecture of annotated XRRA1 transcripts. **S1G,** System-level normal-tissue atlas of XRRA1. The branching diagram organizes XRRA1 expressions across major body systems, tissues and representative cell types, including bone marrow, immune, digestive, integumentary, nervous, respiratory,

bioRxiv preprint doi: https://doi.org/10.64898/2026.04.21.719372; this version posted April 24, 2026. The copyright holder for this preprint (which was not certified by peer review) is the author/funder, who has granted bioRxiv a license to display the preprint in perpetuity. It is made available under aCC-BY 4.0 International license.

urogenital/reproductive and cardiovascular compartments. Box plots at right summarize relative XRRA1 expression across the corresponding tissue and cell-type categories. **S1H,** Cell-line expression atlas of XRRA1. The branching diagram groups cultured cell lines by tissue or lineage of origin, and the accompanying box plots summarize relative XRRA1 expression across the indicated categories. **S1I,** DepMap codependency analyses for XRRA1. Venn diagrams compare positively associated XRRA1 gene sets identified by CRISPR and RNAi screens (54 and 77 genes, respectively) and negatively associated gene sets (47 and 21 genes, respectively). Bar plots show Elsevier Pathway Collection enrichment for the corresponding positive and negative gene sets; bar length indicates −log10(*P*). Gene-level correlation data are provided in Supplementary Tables 7 and 8.

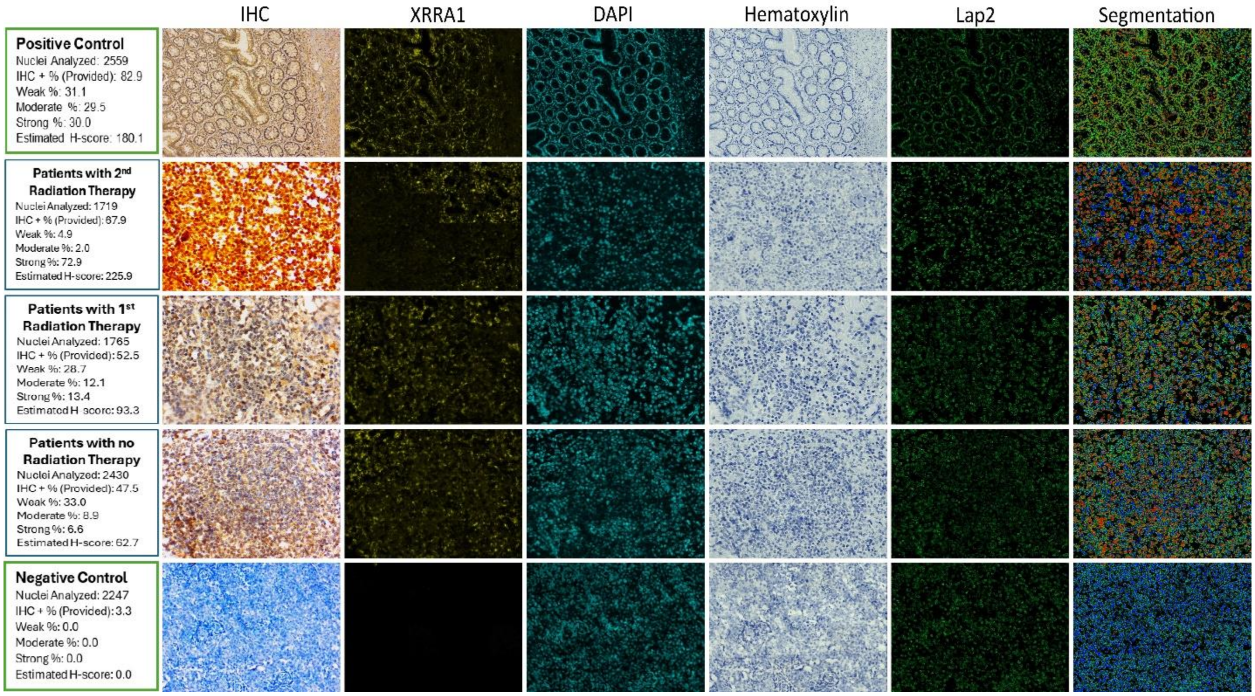


**Supplementary Figure 2: Extended digital pathology analysis of XRRA1 across radiation exposure groups.** Representative XRRA1 immunohistochemistry and DeepLIIF-derived channel decomposition for positive control, second radiation therapy, first radiation therapy, no radiation therapy, and negative control groups. For each condition, the IHC, XRRA1, DAPI, hematoxylin, LAP2, and segmentation panels are shown. Annotation boxes summarize the number of nuclei analyzed, the percentage of IHC-positive cells, the proportions of weak, moderate and strong staining, and the estimated H-score for each representative field.

bioRxiv preprint doi: https://doi.org/10.64898/2026.04.21.719372; this version posted April 24, 2026. The copyright holder for this preprint (which was not certified by peer review) is the author/funder, who has granted bioRxiv a license to display the preprint in perpetuity. It is made available under aCC-BY 4.0 International license.

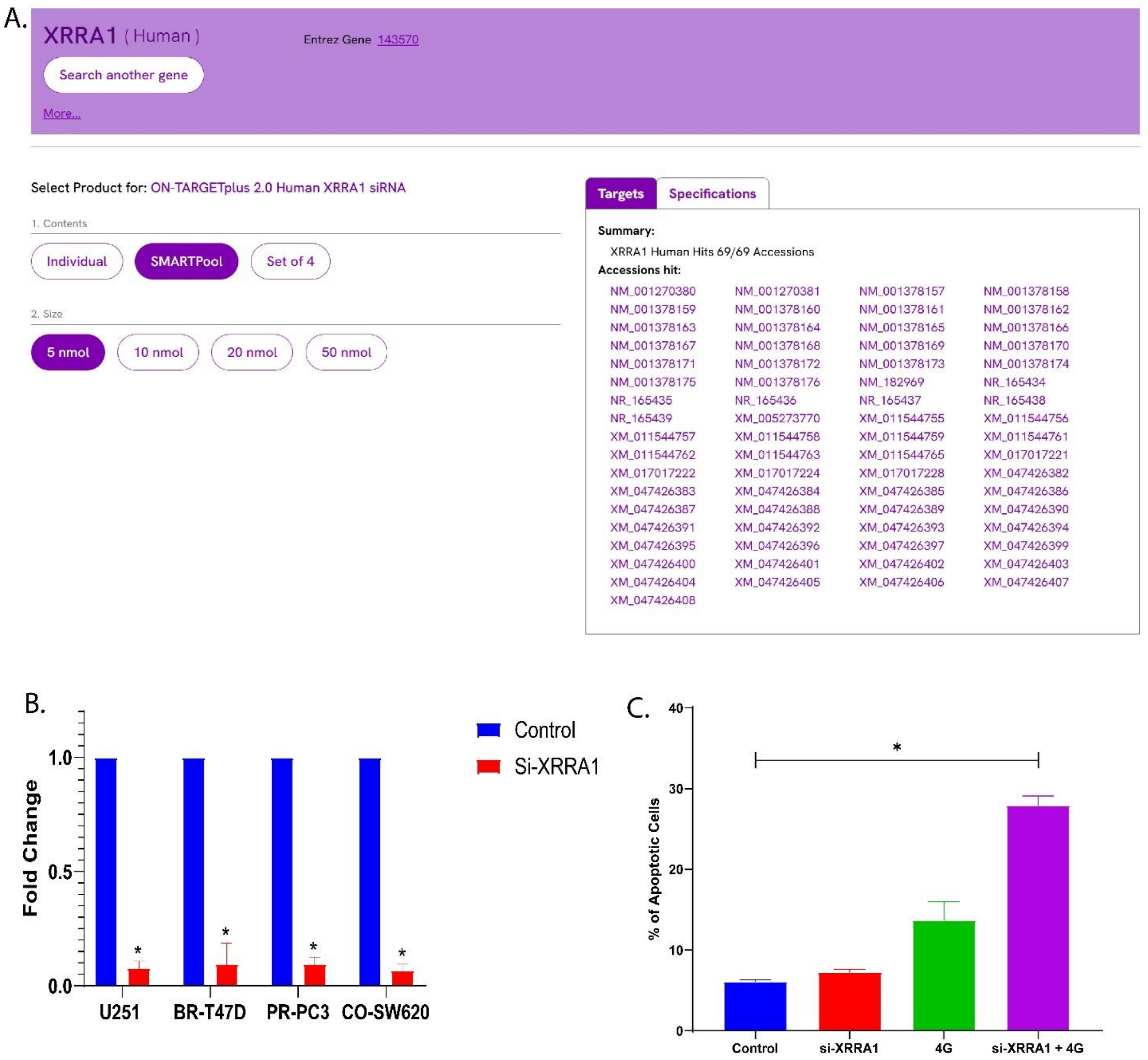


**Supplementary Figure 3: Validation of XRRA1-targeting siRNA and quantification of apoptosis following XRRA1 depletion and irradiation. S3A,** Transcript coverage summary for the ON-TARGET plus 2.0 Human XRRA1 siRNA SMART pool, showing predicted targeting of XRRA1 accessions. **S3B,** Validation of XRRA1 knockdown efficiency in U251, BR-T47D, PR-PC3 and CO-SW620 cells. XRRA1 expression is shown as fold change relative to control, with control normalized to 1. **S3C,** Quantification of apoptotic cells under control, si-XRRA1, 4 Gy, and si-XRRA1 + 4 Gy conditions. Combined XRRA1 silencing and irradiation produced the greatest apoptotic response. Error bars indicate variation among replicate measurements. Statistical significance is indicated in the plot.

bioRxiv preprint doi: https://doi.org/10.64898/2026.04.21.719372; this version posted April 24, 2026. The copyright holder for this preprint (which was not certified by peer review) is the author/funder, who has granted bioRxiv a license to display the preprint in perpetuity. It is made available under aCC-BY 4.0 International license.

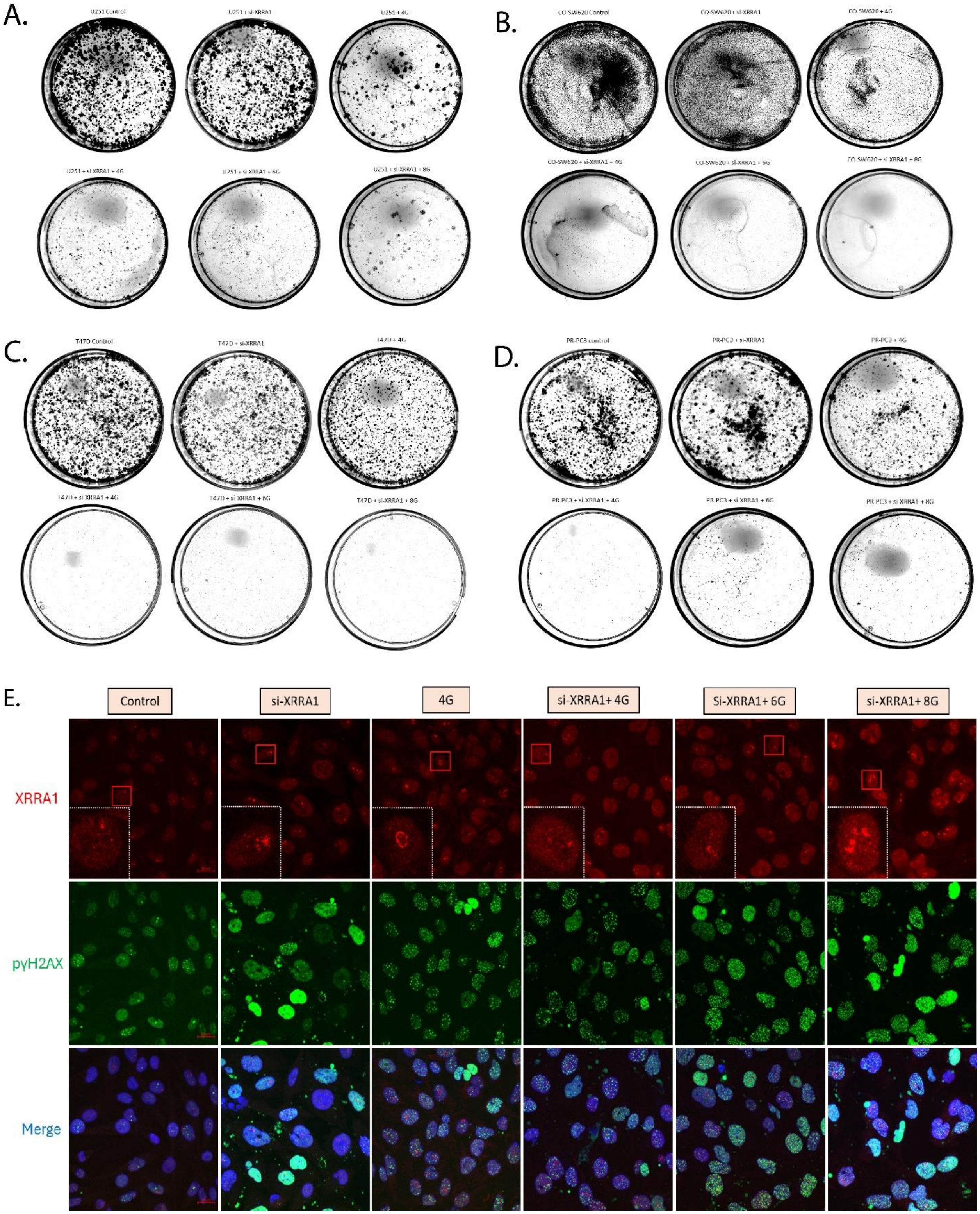

bioRxiv preprint doi: https://doi.org/10.64898/2026.04.21.719372; this version posted April 24, 2026. The copyright holder for this preprint (which was not certified by peer review) is the author/funder, who has granted bioRxiv a license to display the preprint in perpetuity. It is made available under aCC-BY 4.0 International license.

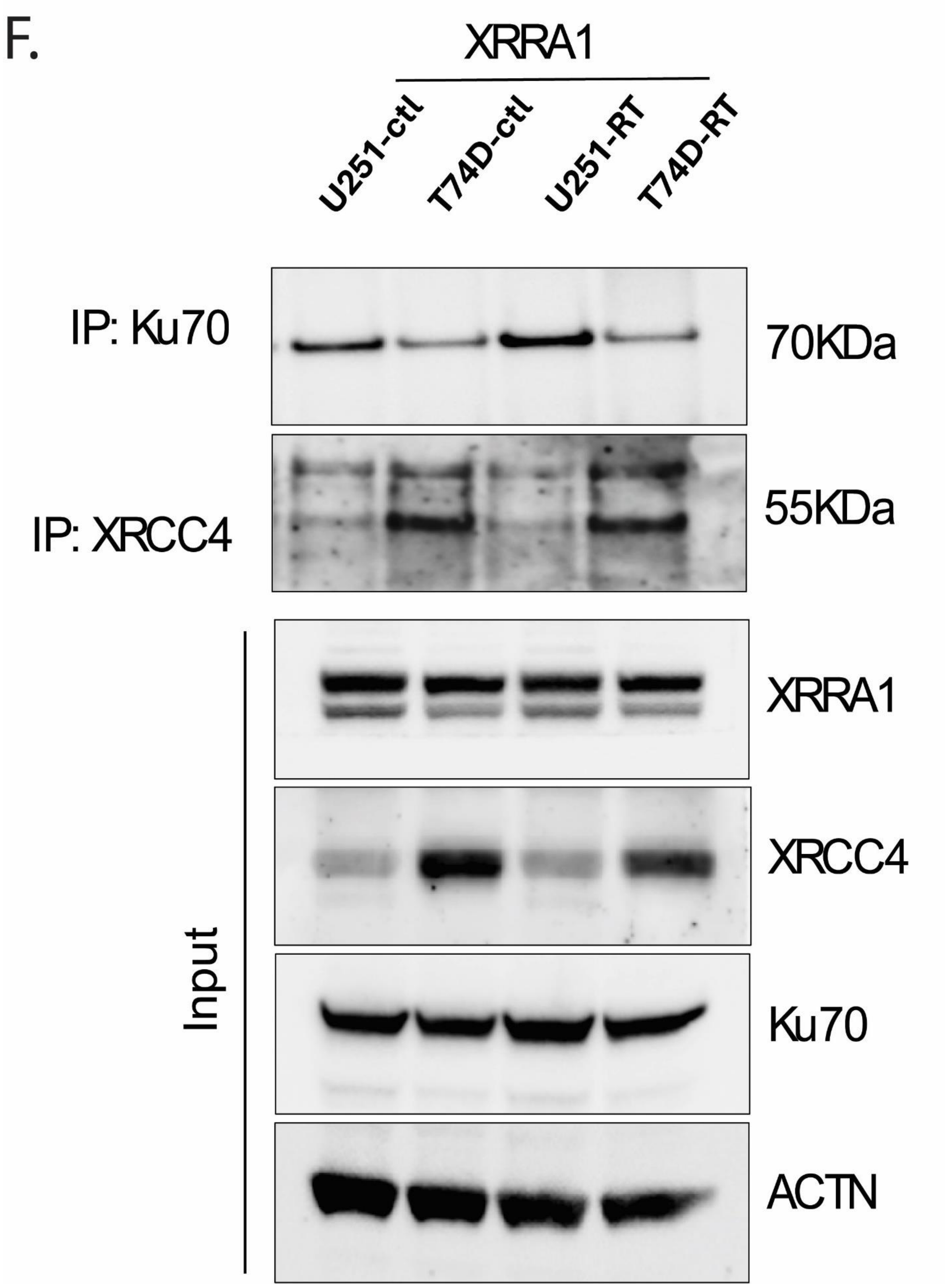

bioRxiv preprint doi: https://doi.org/10.64898/2026.04.21.719372; this version posted April 24, 2026. The copyright holder for this preprint (which was not certified by peer review) is the author/funder, who has granted bioRxiv a license to display the preprint in perpetuity. It is made available under aCC-BY 4.0 International license.

**Supplementary Figure 4: XRRA1 loss reduces clonogenic survival and augments dose-dependent DNA damage signaling. S4A-S4D,** Representative clonogenic plates from U251 (a), CO-SW620 (b), T47D (c) and PR-PC3 (d) cells under control, si-XRRA1, 4 Gy, si-XRRA1 + 4 Gy, si-XRRA1 + 6 Gy, and si-XRRA1 + 8 Gy conditions. Across all four cell lines, XRRA1 silencing enhanced the inhibitory effect of radiation on clonogenic outgrowth, particularly at higher doses. **S4E,** Dose-escalation immunofluorescence images showing XRRA1 (red), p(S139) γH2AX (green), and merged images with nuclear counterstain (blue) under control, si-XRRA1, 4 Gy, si-XRRA1 + 4 Gy, si-XRRA1 + 6 Gy, and si-XRRA1 + 8 Gy conditions. Boxed regions indicate magnified areas. Quantitative analysis is provided in Supplementary Table 13. **S4F,** Co-immunoprecipitation and input immunoblot analysis in U251 and T47D cells under control and irradiated conditions. Upper panels show Ku70 and XRCC4 immunoprecipitates; lower panels show the corresponding input lysates immunoblotted for XRRA1, XRCC4, Ku70 and ACTN.

## Supplementary table legends

**Supplementary Table 1: RT-PCR TaqMan probes list.**

**Supplementary Table 2: Antibodies Lists.**

**Supplementary Table 3: LC–MS/MS protein identification metrics for healthy controls.** Complete LC–MS/MS protein identification dataset for peripheral-blood samples from healthy controls. Columns include protein annotation, molecular weight, isoelectric point, PLGS score, number of identified peptides, number of theoretical peptides, sequence coverage, precursor RMS mass error, number of product ions, digest peptides, modified peptides, product RMS mass error, and product RMS retention-time error. This table underlies the control proteome used for the overlap analysis in Fig. 1a.

**Supplementary Table 4: LC–MS/MS protein identification metrics for chronic myeloid leukemia samples.** Complete LC–MS/MS protein identification dataset for peripheral-blood samples from patients with chronic myeloid leukemia. Columns are as described for Supplementary Table 3. This table underlies the CML proteome used for the overlap analysis in Fig. 1a.

**Supplementary Table 5: STRING interaction dataset for the XRRA1-centred network.** STRING-derived interaction dataset used to generate the XRRA1-centred protein–protein interaction network shown in Fig. 1b. Fields include node 1, node 2, node 1 accession, node 2 accession, node 1 annotation, node 2 annotation, and STRING combined interaction score.

**Supplementary Table 6: Survival statistics for XRRA1 and XRRA1-associated genes.** Gene-wise survival analysis output used for Fig. 1c. Columns include gene symbol, Ensembl gene ID, regression coefficient, standard error, hazard ratio, 95% confidence interval, Z-score and *P* value for XRRA1 and genes within the XRRA1-associated interaction network.

bioRxiv preprint doi: https://doi.org/10.64898/2026.04.21.719372; this version posted April 24, 2026. The copyright holder for this preprint (which was not certified by peer review) is the author/funder, who has granted bioRxiv a license to display the preprint in perpetuity. It is made available under aCC-BY 4.0 International license.

**Supplementary Table 7: Top-ranked CRISPR codependencies associated with XRRA1.** DepMap CRISPR (Chronos) codependency dataset for XRRA1. Genes are ranked by correlation with XRRA1 dependency profiles. The table includes gene symbol, Entrez ID, dataset annotation and correlation coefficient and contains the positive and negative codependency genes used in Supplementary Fig. 1i.

**Supplementary Table 8: Top-ranked RNAi codependencies associated with XRRA1.** DepMap RNAi (DEMETER2) codependency dataset for XRRA1. Genes are ranked by correlation with XRRA1 dependency profiles. The table includes gene symbol, Entrez ID, dataset annotation and correlation coefficient and contains the positive and negative codependency genes used in Supplementary Fig. 1i.

**Supplementary Table 9: XRRA1 immunohistochemical ranking and clinicopathological annotation of the validation cohort.** Ranked summary of XRRA1 immunohistochemical staining across the clinical biopsy cohort. Pages 1–2 list sample rank, sample ID, IHC-positive fraction, and counts of weak (1+), moderate (2+) and strong (3+) cells. Pages 3–4 provide the corresponding H-score, radiation exposure status, radiation therapy cycle, age/sex, UHID, biopsy site, diagnosis, and representative original images. Pages 5–6 show the corresponding DeepLIIF-derived XRRA1, DAPI, hematoxylin, LAP2 and segmentation panels.

**Supplementary Table 10: DeepLIIF-derived digital H-score summary and scoring workflow for representative XRRA1-stained tissues.** Sheet 1 summarizes digital XRRA1 quantification for the representative tissues shown in Fig. 2, including radiation-exposed tissue, tissue without radiation history, placenta positive control, and negative control. Parameters include total nuclei analyzed, XRRA1-positive cells, positive-cell percentage, counts of weak (+1), moderate (+2) and strong (+3) cells, and the calculated H-score (0–300). Sheet 2 describes the digital scoring workflow, including segmentation source, positive-cell definition, intensity measurement from Marker.png, object filtering, pooled intensity thresholds for weak, moderate and strong staining, and the formula used for H-score calculation.

**Supplementary Table 11: Composition of the human XRRA1 ON-TARGET plus SMART pool siRNA reagent.** Composition of the human XRRA1 ON-TARGET plus SMART pool used for knockdown experiments. Columns include gene, species, product type, pool type, siRNA ID, catalogue number, target sequence, molecular weight, and extinction coefficient. The pool comprises four individual XRRA1-targeting siRNAs.

**Supplementary Table 12: Kinase enrichment analysis of XRRA1-associated proteins.** Ranked output of kinase enrichment analysis for XRRA1-associated genes or proteins. Columns include rank, kinase or gene set name, and Z-score. The table highlights the top predicted kinase associations used for interpretation of XRRA1-linked signalling networks.

**Supplementary Table 13: Quantitative microscopy workflow and per-nucleus analysis of XRRA1 and p(S139) γH2AX signals.** Quantitative image-analysis dataset underlying Supplementary Fig. 4e. Method summarizes the analysis pipeline, including dataset composition, treatment conditions, segmentation source, quantified channels,

bioRxiv preprint doi: https://doi.org/10.64898/2026.04.21.719372; this version posted April 24, 2026. The copyright holder for this preprint (which was not certified by peer review) is the author/funder, who has granted bioRxiv a license to display the preprint in perpetuity. It is made available under aCC-BY 4.0 International license.

background subtraction method, positivity thresholds and control-based thresholding rule. Raw Nuclei contains per-nucleus measurements, including nuclear area, background-subtracted XRRA1 and γH2AX intensities, DAPI signal and binary positive calls. Field Summary reports per-field nuclei count, average signal intensities, positive fractions and background values. Condition Summary provides pooled condition-level averages, total nuclei analyzed, positive-cell fractions and fold changes relative to control. Segmentation documents the quality-control procedure used for nuclear segmentation. Because quantification was derived from composite display-normalized PNG panels rather than raw acquisition files, γH2AX trends should be interpreted as more robust than absolute XRRA1 red-channel measurements.
